# Apology in cases of medical error disclosure: Thoughts based on a preliminary study

**DOI:** 10.1371/journal.pone.0181854

**Published:** 2017-07-31

**Authors:** Sonia Dahan, Dominique Ducard, Laurence Caeymaex

**Affiliations:** 1 Neonatal Intensive Care Unit, Centre Hospitalier Intercommunal de Créteil, Créteil, France; 2 Faculté de Médecine, Université Paris Est Créteil UPEC, Créteil, France; 3 Céditec EA 3119, Université Paris Est Créteil UPEC, Créteil, France; Cardiff University, UNITED KINGDOM

## Abstract

**Background:**

Disclosing medical errors is considered necessary by patients, ethicists, and health care professionals. Literature insists on the framing of this disclosure and describes the apology as appropriate and necessary. However, this policy seems difficult to put into practice. Few works have explored the function and meaning of the apology.

**Objective:**

The aim of this study was to explore the role ascribed to apology in communication between healthcare professionals and patients when disclosing a medical error, and to discuss these findings using a linguistic and philosophical perspective.

**Methods:**

Qualitative exploratory study, based on face-to-face semi-structured interviews, with seven physicians in a neonatal unit in France. Discourse analysis.

**Results:**

Four themes emerged. Difference between apology in everyday life and in the medical encounter; place of the apology in the process of disclosure together with explanations, regrets, empathy and ways to avoid repeating the error; effects of the apology were to allow the patient-physician relationship undermined by the error, to be maintained, responsibility to be accepted, the first steps towards forgiveness to be taken, and a less hierarchical doctor-patient relationship to be created; ways of expressing apology (“I am sorry”) reflected regrets and empathy more than an explicit apology.

**Conclusion:**

This study highlights how the act of apology can be seen as a “language act” as described by philosophers Austin and Searle, and how it functions as a technique for making amends following a wrongdoing and as an action undertaken in order that neither party should lose face, thus echoing the sociologist Goffmann’s interaction theory. This interpretation also accords with the views of Lazare, for whom the function of apology is a restoration of dignity after the humiliation of the error. This approach to the apology illustrates how meaning and impact of real-life language acts can be clarified by philosophical and sociological ideas.

## Introduction

Medical care exposes patients to the risk of errors [1]. A medical error (ME) has been defined as an unintended act (either of omission or commission) or one that does not achieve its intended outcome, the failure of a planned action to be completed as intended (error of execution), the use of a wrong plan to achieve an aim (error of planning), or a deviation from the process of care that may or may not cause harm to the patient [2]. When an error causes damage to the patient, it is seen to be unacceptable and calls for redress. For over 20 years now, disclosing the error and the damage caused has been considered necessary by patients, ethicists, and healthcare professionals (HCP) [3–8]; in many countries, national guidelines have been published to encourage such disclosure [8–10]. However, this policy seems difficult to put into practice on a large scale for many reasons: the disinclination to disclose bad news, the fear of blame, or fear of a demand for compensation by the patient affected [8,11]. Medical literature insists on the close attention which must be paid to framing the disclosure of ME, since the way in which the ME is announced and information about the situation communicated is a decisive factor in the experience of both patients and HCP [4–6].

In this context, the act of apology has been described by patients, ethicists and HCP as appropriate and necessary [12,13]. In the medical field as elsewhere, apologies have been shown to help resolve conflict and avoid litigation [14], resulting in positive responses including fewer malpractice suits [15–18].

Depending on the words uttered, a distinction can be made between protective or partial apologies, and admissions or full apologies: protective apologies contain a manifestation of goodwill, such as regret, sympathy and benevolence, whereas apologies admitting error contain a self-critical expression [19]. Over the past twenty years, a growing number of states have adopted “apology laws”. Such laws are protective measures designed to mitigate risk and the fears of doctors that their admission of error might increase the probability of a malpractice suit and financial settlements [8,19–21]. They are intended to encourage those causing injury to apologize, thus expressly ensuring that at least some types of apology cannot be used against them and their institutions in litigation [17]. In practice, “apology laws” and the practices they foster vary between states and countries: all of them protect partial apologies, but some fail to protect full apologies, reading self-critical stances as partial admissions of liability.

A phenomenological and linguistic examination of the notion of apology, as defined by its uses and forms of expression, shows that it contains three conditions: a state of affairs described as an anomaly or abnormality (a breach, shortcoming, deficiency, error, fault or difficulty), a negative assessment of the effects of this state of affairs (unfavourable, not preferable, not desirable, detrimental), and a way out (exoneration, mitigation of the fault, release) for the agent at the origin of the said state of affairs, who may be real or fictitious (seen or imagined). The way out, or issue (from the Old French *eissir*) relates etymologically to the act of emerging from a difficult situation, and this in turn evokes the Latin origin of the verb ‘to excuse’: *excusare*, literally “to be cleared of guilt” in opposition to *accusare*, “to call into question”. It should be noted that the Latin word *causa*, from which comes the French word *chose* (thing), indicates the “cause” or the “trial” then the “affair” or the “thing”. In this way, the act of apology or of excusing oneself fulfils the intention of extricating oneself from a bad situation by addressing a word to others about a state of things, what is at cause, with a view to finding an issue, or way out. The apology allows the person offering it to maintain the relationship without too many impediments, something which is stressed by the philosopher Vladimir Jankelevitch [22]. The apology may be understood, in reference to the interaction theory of the sociologist Erwin Goffman, as one of the techniques for making amends following a wrongdoing inflicted on others. It is part of what he calls *face-work* [23], the action undertaken by a person in order that neither party should lose face. This interpretation accords with the one put forward by Lazare, who has shown that in the field of healthcare, in response to the loss of face brought about by the ME, the restoration of dignity emerges as one of the principal functions of an apology [24].

The act of apology is considered, when uttered using the verbal form of the first person of the present tense: “I apologise” as a performative language act, in reference to John Austin’s theory on speech acts [25]. Utterances which do what they say through the act of saying what they do are called performative utterances by philosophers of language: I perform an act of apology by uttering the apology. Austin classes the verb *apologise* in the family of *comportatives*, which are the performatives leading one to “adopt an attitude” (like *recommend*, *welcome*, *challenge*, etc.). John Searle [26], on the other hand, classes the verb in the family of expressive illocutionary acts (like *thank*, *congratulate*, *deplore*) because they express a psychological state specified by the propositional content of the utterance. *To apologise* is to pronounce the apology while naming it, in the two senses of *pronounce*: to put into words and to proclaim publicly (a judgment, a decision) by virtue of a power or authority. However, in our opinion, the act of apology enters into the more general category of *declaration*, which is a commitment through the word uttered, within a relationship of obligation between the person speaking and others. This is why it is important to explicitly put the apology into words.

Besides these philosophical and linguistic vies on apology, little work has been done to explore the function and meaning of apology in the medical context. Lazare has identified the major healing mechanisms of the apology as sincerity, empathy, forgiveness, trust, dignity and caring [24]. The aim of this study was to explore, through the discourse of doctors, the role ascribed to apology in the communication between HCP and patients when an error with damages is announced, and to discuss these findings using a linguistic and philosophical perspective.

## Data and methods

### Researchers’ perspective

From the perspective of studying communication between doctors and patients, we have carried out a qualitative exploratory study in France, involving doctors. The philosophical perspective was based on a critical study attempting to gain a better understanding of the way doctors view the apology, and indirectly to engender a change in their attitude by encouraging self-examination and awareness of the meaning of the apology in disclosure of a ME.

### Data collection and analysis

Face-to-face semi-structured interviews were conducted with the seven senior doctors working in a single NICU in a university-affiliated general hospital in the Ile de France, between 1 June and 15 August 2012.

The face-to-face interviews were made up of two parts: 1/ a free account by the participant of a situation in daily life in which he/she remembered having given apologies; 2/ an account of another case of apologies being presented in the professional sphere. The feelings about the meaning given to their apology in these situations were the subject of close questioning (similarities and differences between the contexts, intention, facility and verbal expression used).

The meaning of the declaration of error and the way in which it was disclosed were explored, in its different aspects and implications (S1 Appendix. Interview guidelines). The interviews were conducted by a researcher, trained to carry out research interviews (LC). Researchers were not neutral on the possible positive impact of the apology on patients and on their relationship with the physician. Interviews were audio-recorded, anonymous and transcribed by a person sworn to professional secrecy, unconnected to the team. Discourse analysis was used, as it assumes that, socially and historically, language constructs the way we think about things and experience them both ourselves and in relation to others [27]. Analysis was performed by a member of the team, and monitored by a professor of linguistics.

### Ethics statement

Written consent by the participants was obtained in order to take part in the research interview, to agree to an audio recording of the data to be made and for it to be transcribed anonymously. The Ethics Committee named Groupe de Réflexion Ethique of the Centre Hospitalier Intercommunal of Créteil approved this study.

## Results

Between 15 May and 15 July 2012, seven research interviews were carried out (average length 60 minutes; 6 neonatologists and 1 surgeon; 3 women and 4 men; aged 33 to 62 years, mean 46). Analysis allowed four main themes to emerge, which were present in the discourse of the doctors and/or the mother. Verbatim accounts have been added to illustrate the data (Dr1 to 7 for the doctors). Table 1 describes the situations chosen by doctors to report apologies in daily life and in professional life.

**Table 1:** Situations presented during the course of interviews.

Everyday life situations	Medical situations
Being late (apology to wife)	Rectal perforation upon biopsy (Dr1)
Deleting a colleague’s computer data	A baby’s fall from an incubator (Dr2)
Forgetting a mother’s birthday	A dopamine prescription error (Dr3)
Self perceived educational misbehaviour as a father towards his daughter	Bad news concerning another child given to parents in error (by telephone) (Dr4)
Behaving badly towards someone close to you	Complication of digestive surgery (Dr5)
Mother’s apology for her daughter who hurt another child’s face	Wrong medication administered (Dr6)
Request for sister to perform childcare while doctor works	Surgical complication (Dr7)

### Difference between apology in everyday life and apology in the medical encounter

According to the doctors, four factors separated the act of offering apologies in personal life from those offered in the health-care context. First, the consequences of the action were described as more severe in professional than in private life. “*As a doctor*, *the problem is much more serious than anything you encounter in everyday life*.” (Dr2). Secondly, apology was always described as more difficult to sustain where the personal was involved, but more difficult to express and morally significant in professional life. “*In everyday life*, *when the apology is to do with a small problem*, *I have no difficulty apologising*, *so it doesn’t affect me at the level of my self-respect*. *But when it concerns more important problems*, *it costs me more to make the effort to go and apologise*, *I say to myself*: *Phew… I have to go through with this*, *it’s really annoying*, *if I want to continue having relationships with people*, *and also if I want to move on*”. (Dr1) Thirdly, the doctors emphasised that apologies were offered very frequently in personal life, but much more rarely in situations involving professional life. Finally, the professional relationship setting was described as a contract: “*We have a responsibility as part of our work*. *That’s what we’re paid for*” (Dr2), whereas in personal life the apology was addressed either to those who were close (as in a couple) or, on the contrary unknown, involving people who had no particular expectations.

### Place of the apology in the process of disclosure of a ME

The doctors reported having given explanations described as essential when announcing a ME: the chain of causes leading to the error, the consequences for the child’s health and a show of empathy and regret. These elements were part and parcel of the act of apologising. All the doctors reported having uttered words showing feelings of good will and empathy towards the parents: “*I understand that for you this is unacceptable*” (Dr3), as well as of regret: “*I am truly sorry that this has happened*” (Dr5), and of explicit or implicit acknowledgment of personal or collective responsibility. The doctors considered the shared nature of the responsibility should be stressed rather than an individual responsibility: “*I acknowledge that* *we* *have made a mistake*” (Dr4). The explicit wording of the apology played a secondary role for all of them: the word of apology was not uttered explicitly, but was considered implicit in the act of giving explanations. Two doctors stated that they had actually expressed apologies. Finally, two doctors said that they generally gave a description of the strategies put in place to avoid the error being repeated. Two doctors spoke of the possibility of the parents demanding compensation, whereas the five others did not mention it. None of them mentioned the need to hold several interviews with the parents in order to disclose and explain what had happened, nor did they indicate that this was the usual custom.

### Functions and effects of the act of apology

The doctors had to reflect on this question before answering. The effect of the apology was to allow the relationship to continue despite the disaster. “*Apology has a dual aim*: *to enable relationships with people to continue and also to begin a new chapter*” (Dr5), in medicine as in personal life. They saw the apology as fulfilling three other functions, namely to acknowledge responsibility, to inspire forgiveness and to bring about a change of position in the patient/physician relationship. For six of the doctors, the apology by definition included an explicit acknowledgment of responsibility, even if it was made separately. The majority of the doctors said they thought forgiveness could be facilitated by the act of apologising, but could not be obtained by this sole act; they appreciated the personal dimension involved in forgiveness and saw that its timing might not match that of the events themselves: “*I think that relatives are free to forgive or not… there’s no doubt about that… if you ask forgiveness*, *it doesn’t necessarily mean you obtain it… I think that situations where you’re not forgiven are quite rare*, *I think that if this is the case it’s because you have presented things badly”* (Dr1). Two doctors reported that the apology could cancel out the hierarchical rankings and sometimes destabilise the doctor as a result.

### Ways of expressing apology

Both in everyday life and in the healthcare situation, the doctors mentioned different ways of expressing an apology: “*I’m very sorry*” or *“I present my apologies*”; they reported almost never using this latter in a verbal exchange. *“I said*, *“I’m very sorry”*. *Q*: *And you didn’t say*: *I present my apologies*? *R*: *No*. *It’s not an expression I habitually use*. (Dr2). For all the doctors, the expression “*I’m very sorry*” contained a message of apology in itself. For one, these words could carry two meanings, namely the expression of regret and the apology. The expression “*I present my apologies*” or *“I ask you to excuse me*” appeared to be more or even too formal. Only one doctor said he had used it.

## Discussion

This study showed that the apology in the medical encounter has specific characteristics which render it weightier for the HCP than an apology in everyday life. The apology was described as taking place within the extensive process of disclosing the ME, which also contains explanations, regrets, empathy and sometimes ways to avoid repeating the error. Its effects were to allow the patient or proxy-physician relationship, undermined by the ME, to be maintained, responsibility to be acknowledged, the first steps towards forgiveness to be taken, and a less hierarchical doctor-patient or proxy relationship to be created. The ways of expressing the apology were seen differently by the doctors, who mentioned saying “I am sorry” rather than the more formal *I ask you to excuse me*, this way of expressing the apology reflecting regrets and empathy more than a full apology. Apologies described here wee in general protective rather than admissions of error.

This study has enabled us to highlight what unites the meaning of the act of apology with the apology seen as a “language act”, embedded within a situation of communication, where the relationship is affected by the apology—a situation which has arisen because of an event that demands explanation and redress.

In our study, participant doctors assessed errors by their consequences, and only in the most serious cases did they engage in the process of communicating their errors. This is in accordance with what was found in scenarios with parents, where severity of the error was associated with a desire for disclosure [7]. The doctors thus spare themselves a delicate task if the stakes seem to them to be lower. The notion of threat, used in the “face theory”, allows us to understand the hesitations and tensions generated by a situation which justifies apologies. The linguist Catherine Kerbrat-Orecchionni thus states: “To apologise is to threaten one’s own positive face, but not to apologise is to refuse to symbolically make good the wrong committed, and is therefore to threaten the positive face of the other person” [28].

Presenting apologies implies recognition of an adverse event (AE), and an expression of regret, constituting a display of goodwill towards the patent [12]. Only in the case of a full apology does it include an acknowledgment of the responsibility of HCP, team and/or institution towards the affected person, involving the question of collective and/or individual responsibility. Collective responsibility is emphasised here, rather than individual involvement in the process that contributed to the error. The underlying question here is “Who is to blame when an error occurs in healthcare?”. Indeed, in hospitals and healthcare institutions, systemic causes are well known to be contributory factors in generating errors, and even if an individual HCP is involved in one of the steps leading to he error (through negligence, ignorance or bad judgment), the existence of effective barriers preventing the occurrence of errors and/or serious consequences depend on a multilevel state-wide organisation of healthcare [29].

A study in the UK of patients who wish for an apology [30] showed that one third of patients affected by a ME expressed the desire to receive an apology or an explanation. An assurance that something would be done so that the mistake was not repeated in the future was important for them [31]. This was also what emerged from our research.

Moral and ethical, individual and collective responsibility was another feature described in our study. This responsibility is always permeated by a feeling of guilt. Acknowledgment of responsibility by the doctor may have a greater positive effect for patients than the expression of sympathy [32]. The act of apology is present within the explanations about the circumstances and reasons for the event, together with its consequences and possible preventive measures. Designation of fault and acknowledgment of wrongdoing have been shown to be very important healing factors which the apology grants to patients [24]. However, the difference between a preventable AE and an unpreventable AE might not be immediately established, and individual and/or collective responsibility should not be decided early on, without time being taken to investigate what went wrong and to address the issues around preventability and individual and systemic causes. Institutions are asked to put in place a policy for informing families should AE take place. Telling patients about the institution’s information policy enables people to gain a better understanding of the role of the system in the causes of AE and could reduce the desire to punish individuals [33].

The fear of lawsuits, which may be an obstacle to disclosing a ME and delivering an apology, was not central to our interviews or at least did not feature specifically. In our study, doctors rarely reported adopting an explicitly self-critical or guilty stance, but were more likely to express words of goodwill and positive consideration for the patient and parent. Their words “I’m sorry”, without a clear indication of what they were sorry for, leaved the apology partial in most cases. The reason why full apologies were hardly mentioned remains open: was it simply to avoid worrying about this type of risk? The tendency to keep silent because of the risk of proceedings for professional misconduct has been mentioned in many papers [8,19,30]. This attitude accords with the warning contained in the French proverb that “he who apologises condemns himself”.

Finally, the expression of personal criticism or even a guilt feeling should probably not be taken at face value as an admission of responsibility, and used as such in a court context; philosophers have shown that, from a moral point of view, statements expressing self-criticism might reflect the speaker’s sense of having misdirected his efforts to avoid something bad, in a context such as healthcare where agents are deeply invested in avoiding harm to others [19].

Studies have suggested that declaring a ME results in a feeling of respect from the patients, and a lesser demand for redress, along with a desire to settle the dispute. The patients who bring a case to court want their action to prevent a repetition of the event, and they demand an explanation, apologies and a sanction for the sake of example, in order for justice to be done [30], and sometimes humiliation to be reduced [24].

One of the specific features of apologies in the healthcare world is the contractual relationship in which they take place. Our study confirms the data of studies which, among the various forms of resistance by doctors, indicate their difficulty or refusal to admit fallibility, with the consequent damage done to the relationship of trust. The omnipotence of medicine is imaginary but it carries with it the hopes of patients and their loved ones; and a failure of medical power, be it human or technical, is a blow to this contractual relationship. On the medical side there is the presumption that the course of events must follow a normal or normalised process, in accordance with determined criteria set down by medical knowledge, expertise and the experience of the HCP themselves. Medical discourse communicates this presumption, with its share of uncertainty, which the patient does not always grasp and often prefers to forget about. This relationship has been called a “therapeutic alliance” [34]. The way the patient is received by the HCP, and the way the HCP meets his expectations form the basis of the therapeutic alliance. Perceived as a mutual collaboration, this alliance is based on the patient’s “believing expectation”, which refers to the patient’s hope of recovery, the conviction that he is taking the right steps to achieve that end and the power or skill that he assigns to the therapist” [34]. This is why a failure of medicine is a blow to medical power in its ideal representation and, in a way, to the narcissism of its actors. By establishing a new interpersonal relationship, the act of apology can allow a trust which has been weakened or lost to be rediscovered, through the compassion shown by the doctors to which the patients are susceptible. This accords with Lazare’s findings on the psychological effects of the apology: feeling respect for dignity, feeling of being cared for and empathy [15,24].

It is noticeable that, in our study, the doctors, when apologising, appear to favour expressing regret by using the expression: “*I’m sorry*”, a mark of goodwill with the intention of comforting. Regarding the “Disclosure of damage in connection with healthcare”, the Guide for healthcare professionals in France states that: “(…) expressing regret with sincerity and empathy is one of the key elements of the declaration process which contributes to acknowledgment of the damage and to founding a relationship of trust” [9], which might have been partly inspired by Lazare’s findings [24]. It appears, moreover, that the doctor does not expect a formal acceptance of the apology, but rather a sign of understanding, which is not forgiveness. One of the virtues of the apology is to establish a beneficial reciprocity for both parties [9].

This preliminary study has its limits: the number of participants is not high, and their common professional background might mean they have a shared view. It also reflects a Western view of the function and value of apology, both in interpersonal relationships and in its application in the context of healthcare relationships in hospital. Berlinger [35] and Wu [36] point out that, although not universal, the Judaeo-Christian traditions of confession, repentance and forgiveness determine the expectations of individuals in Western societies; apology might be less appropriate in other cultures. This study is dependent on doctors’ statements about their practices and should be confirmed by observations *in situ*. However, real situations are difficult to study for both practical and ethical reasons, and most studies were based on scenarios, discussion groups or surveys ([4,6,37]. Finally, the point of view of relatives or patients was not explored. Exploring the perception of harm on the part of patients and families, where the standard of care has in fact been met, could be a challenging area for apology issues in the future.

The act of apology can be interpreted as a means of regulating human relationships in society. When transposed to the context of hospital procedure, it must lead us to take account of the three spheres of recognition involved, which combine the institutional (public health policy, hospital and its missions, department and its organisation), the inter-personal (courtesy, professional socialisation) and the inter-subjective (empathy, respect). The thinking of all the actors concerned with the question of communication in terms of standards, values and conduct, must be based on political, ethical and psychological considerations. The effect of physicians’ nonverbal involvement during error disclosures on healing mechanisms and the risk of malpractice has recently been demonstrated [38]. In our study, the physicians showed little insight into the issue of apology. None of these doctors had undergone relevant training in this regard. Specific instruction on how to communicate an AE could therefore usefully be based on simulation techniques. A poster or video explaining when and how to word an apology would have the advantage of reaching a wide audience.

In conclusion, this preliminary study, together with the thinking behind it, shows that the apology can be seen as part and parcel of the disclosure of error which comprises explanations, an expression of regret and empathy and the offer of redress. In order to be able to present apologies that have the value of making amends to the person wronged, the doctor has to use a means of expression which is clear: the form of words chosen determines whether or not the apology is understood as a request to be excused and not simply an expression of regret. When it is expressed fully, the apology succeeds as a language act. It can then be an opportunity for a new form of relationship to emerge, one which goes beyond what previously prevailed. From an encounter based on an error it can become the means of bringing together common humanities and shared values. These issues take place in the specific legal and institutional context in which the HCP is working.

## Supporting information

S1 Appendix Interview guidelines. (PDF)
