# Supplementary material for: Apology in cases of medical error disclosure: Thoughts based on a preliminary study
Source: PLoS One. 2017 Jul 31;12(7):e0181854. doi: 10.1371/journal.pone.0181854 (PMC5536280; doi:10.1371/journal.pone.0181854)
Supplement: S1 Appendix — (PDF) [file pone.0181854.s001.pdf]

S1 Appendix. Interview guidelines

Explain what the research consists of, the context in which it is being carried out, the anonymity of the results, ask if the person agrees to participate and consents for the anonymous data to be used for research and teaching.

Ask the person to give an account of a situation, in their personal life, in which they have presented apologies. Then encourage the person to give the same type of free account, but this time involving a situation in which they have revealed an error that has taken place on a patient, and when the issue of an apology has therefore arisen.

1) What feelings did you have towards the other person and yourself when the disclosure was made and apologies offered?
2) What made you approach the person to offer your apologies and what stopped you doing so?
3) Is there a right time to apologise, and how can you know when that is?
4) Do you think that an apology can be a means to establishing a deeper relationship? If so, why?
5) Does being a doctor make the apology different from those apologies that you make in everyday life?
